# *APOE* Alleles and Diet in Brain Aging and Alzheimer’s Disease

**DOI:** 10.3389/fnagi.2020.00150

**Published:** 2020-06-10

**Authors:** Hussein N. Yassine, Caleb E. Finch

**Affiliations:** ^1^Keck School of Medicine, University of Southern California, Los Angeles, CA, United States; ^2^Leonard Davis School of Gerontology and Dornsife College, University of Southern California, Los Angeles, CA, United States

**Keywords:** APOE, Alzheimer’s disease, diet, aging, genetics

## Abstract

The *APOE* gene alleles modify human aging and the response to the diet at many levels with diverse pleotropic effects from gut to brain. To understand the interactions of *APOE* isoforms and diet, we analyze how cellular trafficking of apoE proteins affects energy metabolism, the immune system, and reproduction. The age-accelerating *APOE4* allele alters the endosomal trafficking of cell surface receptors that mediate lipid and glucose metabolism. The *APOE4* allele is the ancestral human allele, joined by *APOE3* and then *APOE2* in the human species. Under conditions of high infection, uncertain food, and shorter life expectancy, *APOE4* may be adaptive for reducing mortality. As humans transitioned into modern less-infectious environments and longer life spans, *APOE4* increased risks of aging-related diseases, particularly impacting arteries and the brain. The association of *APOE4* with glucose dysregulation and body weight promotes many aging-associated diseases. Additionally, the *APOE* gene locus interacts with adjacent genes on chromosome 19 in haplotypes that modify neurodegeneration and metabolism, for which we anticipate complex gene-environment interactions. We summarize how diet and Alzheimer’s disease (AD) risk are altered by *APOE* genotype in both animal and human studies and identify gaps. Much remains obscure in how *APOE* alleles modify nutritional factors in human aging. Identifying risk variant haplotypes in the *APOE* gene complex will clarify homeostatic adaptive responses to environmental conditions.

## Introduction

Circulating lipoproteins have three major roles in lipid binding and transport, that are arguably interdependent and, to a large extent, based on the capacity to exchange lipids among cells and within different cellular compartments. First, lipoproteins provide lipids as a source of cellular energy. Second, lipoproteins supply adrenals and gonads with cholesterol for steroid synthesis pre and postnatally. Third, lipoproteins modulate the innate immune system and susceptibility and response to infecting organisms, whether pathogenic or not. These interdependent roles maintain sufficient energy substrates for reproductive and immune function and to tolerate short bouts of fasting. Lipoproteins provide efficient packaging of lipid-derived energy precursors of cell components. Fatty acids derived from plasma triglycerides are used for energy production by muscles, and, if in excess, lipids are directed into adipocytes for storage. Blood lipid transport is regulated by specific apolipoproteins (apo), lipoprotein receptors, lipolytic enzymes, and transfer proteins, which act in concert to maintain the balance of cholesterol and triglyceride homeostasis in tissues and plasma. Among apolipoproteins, apoE exists in three allelic variants that have multiple influences on human aging. There are emerging subcellular roles of apoE, for its binding to β-amyloid (Aβ) peptides; in mitochondrial metabolism; and as a potential transcription factor in the cell’s nucleus.

The *APOE* gene allelic variants, ε2 (*APOE2*), ε3 (*APOE3*), and ε4 (*APOE4*), differ at two amino acid residues (Table 1). The prevalence of the major allele *APOE3* ranges from 48% to 94%, while the minor *APOE4* allele has a wider range of 3–41% globally (Table 1, Singh et al., 2006; Abondio et al., 2019). *APOE* alleles have a major impact on aging-associated diseases, particularly cardiovascular disease (CVD), stroke, Parkinson’s, lewy body dementia, multiple sclerosis, and late-onset Alzheimer’s disease (AD). The underlying pathologic role of the *APOE* alleles may be understood in terms of its metabolic impact during aging, which has implications for optimizing our diet. These questions are approached by examining basic mechanisms of apoE cell biology relevant to energy metabolism with insights into how adaptive responses to infections could facilitate reproduction, but increase the risk of aging-associated diseases. We also discuss the *APOE* gene cluster and disease risk in different ethnic groups. Lastly, we consider how the effect of apoE on cellular energy preferences can give insights on the failed past clinical trials, and how a more inclusive understanding of apoE could enable the development of novel study designs and drug targets.

**Table 1:** Human APOE polymorphisms and differences by species.

**A: Human APOE polymorphisms and differences by species**			
ApoE Residue (mature peptide)	61	112	158
ApoE2	Arginine (R)	Cysteine (C)	C
ApoE3	R	C	R
ApoE4	R	R	R
Chimpanzee	Threonine (T)	R	R
Mouse	T	R	R
**B: APOE 2 and 4 alleles: prevalence and major characteristics**			
	APOE 4	APOE 2	
Population Frequency*	3-41%	1-38%	
R61—Glu255 domain interactions	Present	Absent	
Protein aggregation	Increased	Lower	
Biochemical Properties	Enhanced binding to lipids	Reduced binding to the LDL-receptor compared with E3 and E4	
Lipid Metabolism	Hypercholesterolemia Hypertriglyceridemia	A small percentage have hypertriglyceridemia
BMI and disease association	Lower BMI, particularly with aging	Greater BMI with homozygotes	
Insulin resistance	Increased	Lower	
Chronic Inflammation	Enhanced response to inflammation	Lower response to inflammation	
Brain amyloid plaque accumulation	Increased	Lower	
Alzheimer’s disease risk	Increased	Protective	
Blood-brain barrier integrity	Compromised	Not studied	
Vascular system	Increased atherosclerosis	Mixed. Protects against heart disease, but increases risk of intracranial hemorrhages	

*Abondio et al. (2019): data of 1000 Genome Project integrated with Singh et al. (2006).

### ApoE Structure and Function

ApoE lipoproteins have crucial roles in cholesterol and lipid flux between tissues during fasting and postprandially. As an exchangeable apolipoprotein, apoE shuttles between larger lipid-containing VLDL particles and the smaller protein-containing HDL particles (Blum, 1982). On VLDL, apoE promotes VLDL clearance and lipid loading into cells *via* apoE receptors such as the LDL receptor (LDLr) family. Following lipolysis, apoE is exchanged to HDL particles, which have a longer half-life and more complex functions. VLDL is catabolized faster than HDL and has a higher affinity to surface apoE receptors. These biochemical properties have a major impact on VLDL and HDL metabolism, and affect the distribution of lipids carried by these particles in different tissues, discussed below.

The differing presence of cysteine vs. arginine at sites 112 and 158 of apoE affects its binding of lipids and receptors. ApoE3, the most common isoform, contains cysteine and arginine at positions 112 and 158, respectively (Table 1). ApoE2 has two cysteines and apoE4 two arginines at these positions. For high-affinity binding, apoE must be bound to phospholipids or lipoproteins. ApoE4 has greater lipid binding affinity than apoE3 and apoE2, which has a major effect on apoE’s functions. Lipid-free apoE does not bind with a high affinity to LDL receptors. Glycosylation and sialylation of apoE affect the binding of apoE to HDL (Marmillot et al., 1999). In cerebrospinal fluid, apoE is heavily sialylated compared to plasma (Hu et al., in press). The sialylation is at the C terminus and appears to differ by isoform (Flowers et al., 2020), although much more work is needed to address the isoform-specific effects of sialylation on apoE lipid binding and function in the brain.

Two key properties of apoE4 that explain its greater lipid-binding properties are a domain interaction and reduced stability relative to apoE2 and apoE3 (Dong and Weisgraber, 1996; Morrow et al., 2000). The term ‘domain interaction’ refers to an interaction between R61 in apoE4 with the acidic Glu255, which is mediated by the positively charged arginine at position 112. This in part explains the preferential binding of apoE4 to large VLDLs, whereas apoE3 and apoE2 prefer smaller HDLs (Weisgraber, 1990). This binding property results in more apoE molecules per lipid particle than apoE3 and apoE2 (Gong et al., 2002). The higher density of apoE molecules per lipid particle enhances apoE4’s affinity to LDL receptors. Per apoE molecule, apoE3 and apoE4 bind to LDL receptors with similarly high affinity, while the binding of apoE2 is 100-fold lower (Weisgraber et al., 1982). Mouse apoE, like apoE4, contains the equivalent of R112 and Glu255, but lacks the critical R61 equivalent (it contains T61). The importance of T61 to domain interactions was shown in mice by targeted mutagenesis and replacement of T61 with R61 (Dong and Weisgraber, 1996; Raffai et al., 2001). The engineered T61 to R61 apoE lost the wildtype binding preference for HDL and enhanced its affinity to VLDL (Raffai et al., 2001). Moreover, the R61 mouse had a 40% higher level of brain amyloid peptides than C57BL/6, together with spatial memory deficits (Adeosun et al., 2019). The chimpanzee apoE resembles mouse apoE at T61, which predicts apoE3-like lipid binding, despite its apoE4-like R112 and R158 (Finch, 2010). However, chimpanzee apoE differs from humans in other amino acids, e.g., four of the eight residues that showed positive selection in the human lineage are within the lipid-binding C-terminal region (Vamathevan et al., 2008).

ApoE isoforms also differ considerably in the conformational stability of their N-terminal domains: apoE4 is the least resistant to thermal and chemical denaturation, apoE2 is the most, and apoE3 has intermediate resistance. The folding intermediates of apoE4 present a core alpha-helical structure with increased beta-structure and an increased hydrodynamic radius, promoting the “molten globule” state. This semi-folded structural state enhances the binding of apoE4 to larger lipid-containing particles in plasma and Aβ deposits in the brain (Chetty et al., 2017). Importantly, the molten globule state favors the aggregation of monomeric and poorly lipidated apoE. At the low pH of endosomes, apoE4 is more favored than apoE3 to form a molten globule with its increased binding affinity to lipids (Morrow et al., 2002). ApoE aggregation has a role in neurodegenerative diseases such as AD (Rawat et al., 2019), predisposing the aggregation of interacting proteins, e.g., seeding of Aβ fibrils.

### The Importance of ApoE Recycling to Cellular Bioenergetics

ApoE is unique among the apolipoproteins in its ability to recycle in and out of cells, with minimal intracellular degradation (Farkas et al., 2003). After intracellular uptake of apoE containing lipoprotein particles, e.g., in liver cells, the internalized lipids are dissociated from apoE into late endosomal compartments, followed by recycling of apoE through early endosomes and its re-secretion within or into HDL particles. In liver cells, the recycling of apoE is stimulated by smaller HDL particles and is associated with cholesterol efflux to HDL (Heeren et al., 2003).

**Figure 1:**
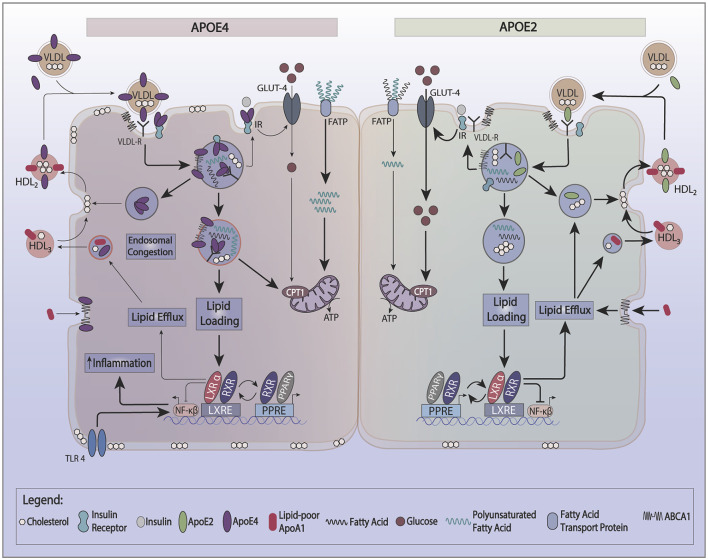
Effect of apoE recycling and aggregation on glucose and lipid metabolism. ApoE recycling controls the expression of several cell surface proteins, such as the insulin receptor (IR), ATP bindingcassette 1 (ABCA1), or lipoprotein receptor-related protein 1 (LRP-1). The formation of smaller HDL3 by ABCA1 stimulates apoE recycling. In the circulation, apoE exchanges between HDL and VLDL. Upon lipid loading, the expression of apoE, ABCA1, and ABCG1 is induced *via* the PPAR/LXR/RXR system to facilitate lipid storage or oxidation and formation of HDL. ApoE4 is prone to aggregate in endosomes trapping interacting proteins such as IR and ABCA1. ApoE4’s switches the cellular energy preference from glucose to polyunsaturated fatty acids, and associates with lower ABCA1 activity and greater cell membrane cholesterol. Greater cell membrane cholesterol enhances TLR4 signaling and activates the inflammasome. ApoE4 also decreases the activation of PPARγ contributing to lower insulin sensitivity and utilization of glucose as a source of ATP.

One of the characteristics of apoE4 is its lower recycling capacity, which likely results from its greater affinity for lipid binding. Indeed, HDL induced cellular recycling of apoE4 is much weaker than other apoE isoforms. This property decreases cholesterol efflux (Heeren et al., 2004) and enriches the cell membrane with cholesterol. The lower pH in early endosomes promotes apoE aggregation and contributes to its reduced secretion from cells. ApoE forms complexes with several surface proteins (the apoE interactome) such as LRP1, ABCA1, ApoER2, and the insulin receptor (IR). ApoE’s propensity to co-aggregate with these proteins in endosomes reduces the plasma membrane levels of these cell surface proteins (Figure 1).

Recycling of apoE appears to depend on the expression of the LDL receptor (LDLr; Fan et al., 2011) and the activity of ATP binding cassette 1 (ABCA1; Rawat et al., 2019). ABCA1 functions to lipidate apoA-1 and apoE, forming small nascent HDL particles. While ABCA1 activity is not required for apoE recycling (Braun et al., 2006), it can indirectly enhance apoE recycling through mediating the formation of smaller HDL particles (HDL3) which directly stimulate apoE secretion and recycling.

Reduced recycling of apoE4 affects its cellular energy source preferences. ApoE complexes with the IR and reduced apoE recycling trap the IR in the endosomes away from the cell surface (Zhao et al., 2017). This reduction in IR surface expression causes reduced utilization of glucose to generate ATP and promotes fatty acid oxidation. Neuronal cell lines expressing *APOE2* have more hexokinase, a critical enzyme of glycolysis, which yields a more efficient production of energy from glucose. Neuronal cell lines expressing *APOE4*, on the other hand, have lower hexokinase activity (Wu et al., 2018). Also, human *APOE2* expressing immortalized astrocytes have a 2.5-fold greater glucose uptake, while *APOE4* astrocytes have half the glucose uptake capacity of *APOE3* (Williams et al., 2020). The effect of genotype on APOE-TR mice models is complex and dependent on the dietary background. Under a chow diet (5% fat), the brains of 15-months old APOE4 targeted replacement (TR) mice show an increase in 18-FDG glucose brain uptake by PET (Venzi et al., 2017). By fMRI, older APOE4-TR mice on a chow diet show increased hyperexcitability at the entorhinal cortex, together with changes in metabolism suggestive of enhanced mitochondrial oxidation activity (Nuriel et al., 2017a). In contrast, APOE4-TR mice on a high-fat diet (60% fat) demonstrate a different phenotype: lower glucose uptake in the frontal lobe, and hippocampal tissue insulin resistance (Zhao et al., 2017; Johnson et al., 2019). Following a high fat but low omega-3 diet, APOE4-TR mice demonstrate lower plasma and adipose tissue omega-3 levels with greater expression of fatty acid-binding proteins (FABPs) and liver carnitine palmitoyl transferase1 (CPT1) than APOE2-TR mice in both liver and adipose tissues. These changes promote greater oxidation of polyunsaturated fatty acids (PUFAs; Conway et al., 2014). Additional features of *APOE4* include changes in lipid droplets. Lipid droplets are dynamic organelles that play a role in various metabolic diseases and appear in many cell types, including brain cells. Lipid droplets are increased in neurodegenerative diseases such as AD (Hamilton et al., 2015). *APOE4* astrocytes display an increase in the number of smaller lipid droplets compared to E3 astrocytes, with a preference for greater endogenous fatty acid oxidation and have a greater susceptibility to CPT1 inhibition (Farmer et al., 2019).

**Figure 2:**
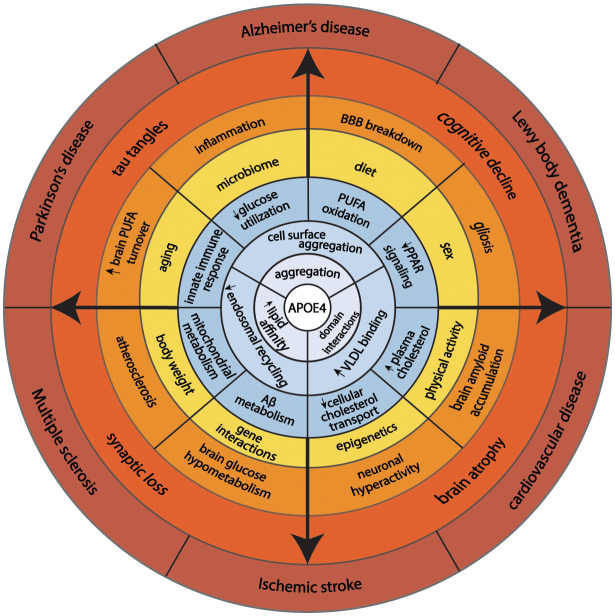
This model illustrates how fundamental structural properties of apoE4 (domain-interactions, greater affinity to lipid binding, and self-aggregation) alter cellular biology promoting endosomal congestion, cell surface aggregation with other proteins, and association with larger lipid-containing particles in the circulation. These biochemical features associated with changes in cellular energy preferences, cholesterol transport, immune response, and Aβ metabolism. Complex interaction with aging, sex, diet, physical activity, and genetics predispose *APOE4* carriers to aging-associated diseases.

Reduced recycling of apoE4 also affects cellular cholesterol metabolism. ApoE4 traps ABCA1 in endosomes away from the cell surface (Rawat et al., 2019). Reduced ABCA1 activity results in lower cholesterol efflux to HDL and redistributes cholesterol to cell membranes. In macrophages, greater membrane cholesterol is associated with activated TLR4 signaling, which, in turn, induces NFkB and inflammatory gene responses (Westerterp et al., 2013). A greater distribution of cholesterol to the neuronal plasma membrane promotes BACE1 expression and APP processing to produce more Aβ-amyloid peptide (Cui et al., 2011). In microglia and astrocytes, less cholesterol efflux reduces Aβ degradation (Lee et al., 2012; Rawat et al., 2019). Another effect of reduced ABCA1 activity is lower apoE lipidation. Since poorly lipidated apoE4 is more aggregation-prone than lipidated apoE4 (Hubin et al., 2019), lipid-poor apoE4 traps ABCA1 in endosomes and lowers ABCA1 activity. This process may be reversed by enhancing ABCA1 activity by small HDL to stimulate the recycling of apoE (Rawat et al., 2019). As noted above, lipidated apoE is less aggregation-prone. Therefore, enhancing the ABCA1 activity provides a therapeutic approach to stimulate the recycling of apoE4 out of endosomes and restore the function of cell surface expression of membrane proteins that interact with apoE. This could be a promising therapeutic target to modulate apoE4’s effects on cellular energy preferences. Figure 2 gives a model that integrates the basic biology of apoE with disease risk.

### Genetic Regulation APOE Expression Through the PPAR-LXR-ApoE System

The genetic control of *APOE* expression differs by cell type and is tightly linked to the lipid loading of cells (Laffitte et al., 2001). ApoE, ABCA1, and ABCG1 proteins are highly induced in lipid-loaded cells, including hepatocytes, adipocytes, and astrocytes, to facilitate lipid exchange and utilization. The nuclear receptors LXRα and LXRβ mediate the effect of lipid loading on the expression of apoE, ABCG1, and ABCA1. The relation of apoE4 expression to PPARγ activity may underlie the association of *APOE* gene expression with inflammatory and cellular energy utilization preferences. As observed for LXRs, the activation of PPARγ can induce gene expression for both ABCA1 and *APOE* (Chawla et al., 2001). Reciprocally, PPARγ can induce the expression of LXRα, thereby creating a metabolically linked cycle that increases apoE expression. Induction of PPARγ activity sensitizes glucose uptake by insulin, stimulates adipogenesis, and dampens the inflammatory response (Leonardini et al., 2009). However, the PPAR-γ signaling pathway may be blunted in *APOE4* (Wu et al., 2018) by presently obscure mechanisms. This complex relationship implies that the interventions that enhance PPARγ signaling are less effective in *APOE4* carriers. This concept has implications for pharmacological and lifestyle interventions that work through PPARγ signaling pathways as discussed below.

### Effect of APOE4 on Triglyceride and Cholesterol Metabolism

**Figure 3:**
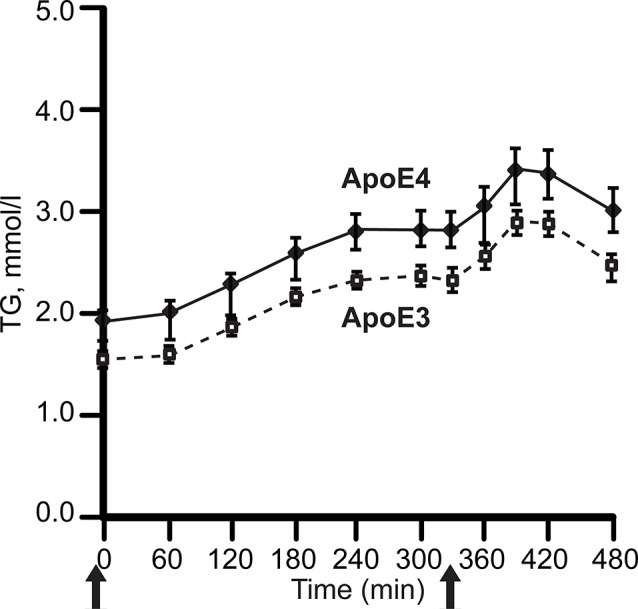
*APOE* alleles and post-prandial plasma triglyceride levels (TG). Plasma was sampled after two meals in healthy adults, ages 20–70 years. with BMI 19–32 kg/m^2^; *APOE3* homozygotes, *N* = 142; *APOE3/4* heterozygotes, *N* = 65. After fasting overnight, subjects received breakfast (49 g fat, *t* = 0) and lunch (29 g fat, 330 min), solid arrows. The *APOE4* excess of plasma TG did not differ by age. For clarity, the graph omits *APOE2* carriers, whose triglycerides was close to *APOE4*. Redrawn and simplified from Carvalho-Wells et al. (2012).

*APOE4* carriers display both hypertriglyceridemia and hypercholesterolemia (Dallongeville et al., 1992; Carvalho-Wells et al., 2012). In contrast, *APOE2* carriers have lower LDL cholesterol (LDL-C) levels, while some *APOE2* carriers have hypertriglyceridemia. Postprandial lipidemia, for example, is elevated in *APOE4* carriers, (Figure 3; Carvalho-Wells et al., 2012).

The mechanism for hypertriglyceridemia in *APOE4* may involve its stronger binding to VLDL, which decreases lipoprotein lipase mediated lipolysis (Li et al., 2013). A major mechanism for hypercholesterolemia with *APOE4* is through the sequestration of apoE proteins on the hepatic cell surface. The lower LDLR affinity of apoE2 increases plasma apoE levels (Blanchard et al., 2018). The elevated plasma apoE2 transfers onto VLDL, which then facilitates LDLR and heparan sulfate proteoglycans (HSPG) mediated uptake without sequestration of smaller LDL particles. In contrast, apoE4 is more confined to the hepatic cell surface than apoE2 (Altenburg et al., 2008). The high LDLR affinity of apoE4 on VLDL particles keeps it bound to the hepatic surface, which explains the increase in degradation of apoE4 and lower plasma apoE4 levels. The sequestering of VLDL particles in *APOE4* carriers on the hepatocyte surface exposes them to lipases for subsequent conversion to remnants and LDL (Altenburg et al., 2008), providing a mechanism for the greater levels of LDL-C with *APOE4*.

### APOE4 and Adipocytes

ApoE is highly expressed in adipocytes, where it modulates adipocyte lipid flux and mediates the effects of PPAR-γ agonists on lipogenesis (Huang et al., 2006). Endogenous adipocyte apoE is important for regulating cell size, triglyceride content, adipose-specific gene expression, and inflammation. Adipocytes isolated from apoE-knockout (−/−) mice are smaller, show decreased adipogenic gene expression, and have lower triglyceride and fatty acid content than wildtype (Huang et al., 2006). In humans and APOE-TR mice, the *APOE4* allele is associated with lower BMI but greater aspects of the metabolic syndrome manifested in elevated plasma glucose and insulin (Fallaize et al., 2017), particularly in obese *APOE4* carriers as discussed below. These changes may be attributed to the inhibitory effects of *APOE4* on PPAR-γ signaling (Wu et al., 2018). Interactions of diet and *APOE* alleles were shown for APOE-TR mice (Arbones-Mainar et al., 2010). After feeding a western-type high-fat diet for 12 weeks, APOE4-TR mice developed greater impaired glucose tolerance than APOE3-TR mice. Treatment with the anti-diabetes drug rosiglitazone (1.5 mg/g body weight) for an additional 4 weeks improved glucose tolerance only in APOE3 mice, but improved plasma lipid profiles for both APOE3 and APOE4-TR mice. Induction of adipogenesis and lipogenesis was severely blunted in adipose tissues, but not in the livers, of APOE4-TR mice. Consequently, lipids were redistributed to the liver, causing marked steatosis in these mice. Furthermore, *APOE* alleles show the sex-specific effects of a high-fat diet on metabolic measures. Male APOE4-TR mice were more susceptible than male APOE3-TR mice to metabolic disturbances, including visceral adipose tissue accumulation and glucose intolerance following 12 weeks of an HFD, while female APOE3 and APOE4-TR mice had similar metabolic responses (Jones et al., 2019).

The mechanism for these observations may result from the failure of thiazolidinediones to stimulate PPARγ activation and adipocyte differentiation in preadipocytes and embryonic fibroblasts isolated from APOE4 vs. APOE3-TR mice. Since adipose tissue expression of apoE is modulated by PPARγ agonists, the increase in apoE4 gene expression inhibits PPARγ signaling effects on adipogenesis (Yue et al., 2004). This coregulation of insulin sensitivity and *APOE* gene expression makes *APOE4* carriers resistant to mechanisms of enhancing insulin sensitivity through liver X receptor and PPARγ in adipocytes (Arbones-Mainar et al., 2010). These findings help explain why APOE4-TR mice on fatty western-type diets gain less body weight and adipose tissue than those with APOE3-TR mice, despite having larger adipocytes (Arbones-Mainar et al., 2008). The inability to form new adipocytes in *APOE4* together with a greater predisposition to PUFA oxidation has implications for the storage and distribution of lipids. For example, APOE4-TR mice have 40% lower adipocyte docosahexaenoic acid (DHA) content compared to APOE3-TR mice on an omega-3 deficient diet (Conway et al., 2014), which may explain the vulnerability of human *APOE4* carriers to an omega-3 deficient diet. *APOE4* associates with reduced adipocyte insulin signaling manifested by less weight gain and impairment of glucose tolerance during a western diet (Arbones-Mainar et al., 2008, 2016). These *APOE4* properties have implications toward dietary recommendations with aging: a shift from a glucose to fat as a source of brain energy and vulnerability to a low omega-3 diet.

**Figure 4:**
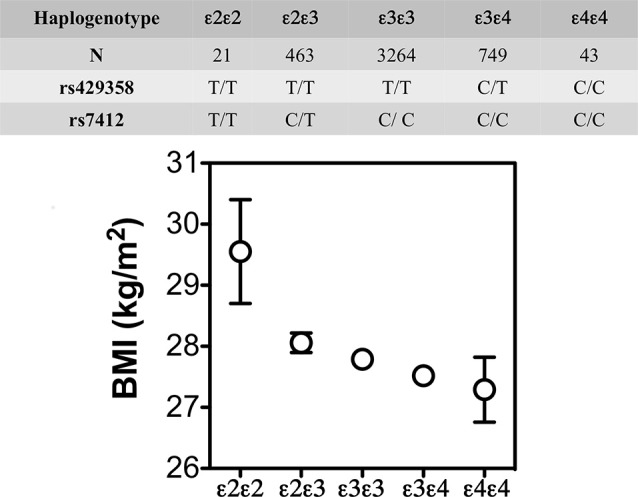
BMI (mean ± SE) by *APOE* isoforms. In the Spanish Aragon Workers Health Study (*n* = 4,881) *APOE* isoforms were associated with body mass index (BMI) in rank order of APOE4 < APOE3 < APOE2. *APOE2/E2* carriers (*n* = 21) had a greater BMI than the other isoforms. Adapted from Tejedor et al. (2014).

The lower weight gain and greater insulin resistance with *APOE4* were also reported in some, but not all, human studies. For example, in the Atherosclerosis Risk in Communities study (*N* = 15,000 individuals; Volcik et al., 2006) and the Spanish Aragon Workers Health Study (*N* = 4,881; Tejedor et al., 2014) *APOE* isoforms were associated with body mass index (BMI) in rank order of *APOE4* < *APOE3* < *APOE2*. The later also showed that *APOE2/E2* carriers (*n* = 21) had a greater BMI than the other isoforms (Figure 4). Obese *APOE4* men had greater measures of IR (Elosua et al., 2003). These findings were not seen in non-obese *APOE4* carriers or individuals with other *APOE* genotypes. Besides, they were also sex-specific: only men showed these *APOE* allele associations with obesity. These studies show that *APOE2* decreases the risk of metabolic syndrome but not higher BMI, while *APOE4* increases the risk of metabolic syndrome, and that these effects may be sex-specific.

Cognitive functions are influenced by complex interactions of *APOE* genotype with obesity that differ by sex, age, and co-inherited gene variants (Table 2). Midlife obesity was associated with an increased risk of late-onset AD in *APOE4* carriers (Ghebranious et al., 2011). Also, in a longitudinal cohort of the Framingham Heart Study, an increase in the waist to hip ratio from ages 40–79 was associated with impaired executive function and increased white matter hyperintensities (mean age 61 ± 9 years; Zade et al., 2013). These findings differ later in life. In a longitudinal population-based sample of 4,055 participants interviewed at 3-year intervals from 1993 to 2012, obesity in older *APOE4* carriers was associated with slower cognitive decline (Rajan et al., 2014). The Prospective Population Study of Women (PPSW) in Sweden showed an increased risk of cognitive decline with later life weight loss. This systematic sample of 1462 women born between 1908 and 1930 and aged 38–60 years at baseline examined several decades later for the incidence of dementia in relation to BMI, and *APOE4* allele status. Women carrying *APOE4* who experienced greater weight loss later in life had a higher risk of dementia (Backman et al., 2015). Taken together, these findings suggest that obesity may be protective against cognitive loss in older *APOE4* carriers, but not during middle life. We suggest an age-specific complex interaction between *APOE4* and body weight on vascular risk on cognitive outcomes. Younger obese individuals with *APOE4* have an increased risk of metabolic and vascular disease that negatively affects cognitive functions later in life. In contrast, obesity in older *APOE4* carriers may provide fatty acids as brain energy fuel with an opposing effect.

**Table 2:** The interaction between aging, obesity, *APOE4* with cognitive outcomes.

Author	Design	Age	ApoE4 effect
Ghebranious et al. (2011)	Cross-sectional (302 controls, APOE4 18% and 150 AD cases, APOE4 60%)	BMI at age 50. Age of assessment was 87 in cases and 78 in controls	Obesity at age 50 was associated with increased AD risk in *APOE4* carriers
Zade et al. (2013)	Cross-sectional (general population, *n* = 1,969, 21% APOE4 carriers)	40–79, mean age 61	*APOE4* with greater waist to hip ratio was associated lower measures of executive function and white matter hyperintensities
Rajan et al. (2014)	Longitudinal (*n* = 4,055), APOE4 34%. Interviewed at 3-year intervals for 19 years	Age > 65	Obesity and *APOE4* showed slower cognitive decline
Backman et al. (2015)	Longitudinal *N* = 559; trajectories of BMI for 37 years	Age > 37	*APOE4* was associated with a steeper decline in BMI and greater AD incidence

### APOE Genotype and Sex

Some studies indicate a sex-*APOE* interaction on the brain. For example, in AD brains, the *APOE4* allele shows male excess for cerebral microbleeds, a marker of small vessel disease, which is opposite to the female excess of plaques and tangles (Finch and Shams, 2016). Sex differences in *APOE4*-associated AD risk appear at younger ages. For example, in an analysis of research studies in the Global Alzheimer’s Association Interactive Network with data on nearly 58,000 participants, men and women with the *APOE* ε3/ε4 genotype had nearly the same odds of developing AD from age 55 to 85 years. However, for a subgroup between the age of 65 and 75, the risk of AD was greater in women than men (Neu et al., 2017).

### APOE Genotype and the Immune System

Macrophage production of apoE regulates its inflammatory properties (Baitsch et al., 2011). The expression of apoE convertsthe macrophage phenotype from a pro-inflammatory to an anti-inflammatory phenotype. Exposure of apoE receptor-expressing macrophages to apoE led to the expression and/or the liberation of several markers (i.e., Arg-1, Fizz1/Relm, SOCS3, IL-1RA). Second, functional characteristics of macrophages exposed to apoE included reduced migration and attenuated ROS generation and cytotoxicity, as well as up-regulated phagocytic activity (Baitsch et al., 2011). In the brain, binding of lipidated apoE to microglia’s LRP1 receptor inhibits neuroinflammation (Brifault et al., 2017). However, there is evidence to support differences in the inflammatory response based on *APOE* genotype.

A unique study compared normal and clinical patients and TR mice for associations of *APOE* alleles with inflammatory responses (Gale et al., 2014). In humans, *APOE4* increased serum interleukin (IL)-1β, IL-6, IL-8, IL-10, IL-17, and tumor necrosis factor-α (TNF-α) responses to LPS (endotoxin) using *in vivo* and *ex vivo* assays. *APOE4* carriers with severe sepsis had more thrombocytopenia. Correspondingly, APOE4-TR mice had greater responses IL-6 and TNF-α (the only cytokines assayed). In a murine monocyte-macrophage cell line stably transfected to produce equal amounts of human apoE3 or apoE4, LPS stimulation in apoE4-macrophages showed higher and lower concentrations of TNF-α (pro-inflammatory) and IL-10 (anti-inflammatory), for mRNA and protein levels. Furthermore, apoE4-macrophages had enhanced the transactivation of the key redox-sensitive transcription factor NF-κB (Jofre-Monseny et al., 2007). One mechanism for *APOE4* associated higher inflammatory responses may relate to the increase in TLR4 activity by greater cell membrane cholesterol distribution from lower ABCA1 activity (Westerterp et al., 2013), as discussed above.

Chronic inflammation increases AD risk with *APOE4*. Data from 2,656 members of the Framingham Heart Study offspring cohort examined longitudinal measures of serum C-reactive protein (CRP) in relation to the diagnoses of incident dementia, including AD, and brain volume. *APOE4* coupled with chronic low-grade inflammation, defined as a CRP level of 8 mg/L or higher, was associated with an increased risk of AD compared to *APOE4* without inflammation, and *APOE2* and *APOE3* with chronic inflammation (Tao et al., 2018).

As the ancestral human isoform, *APOE4* may be beneficial in infectious environments with high pathogen loads (Trumble and Finch, 2019). Children carrying *APOE4* in Brazilian slums, are more resistant to diarrhea and have better cognitive development (Oriá et al., 2010), while adult Tsimane farmer-foragers in Bolivia with *APOE4* have better cognition during high parasitemia (Trumble et al., 2017). Moreover, in the highly infectious environment of rural Ghana, APOE4 carriers showed survival advantage as older adults and children, suggesting reproductive advantage (van Exel et al., 2017). *APOE4* was also protective of HCV infection (Price et al., 2006). These findings are shown for APOE-TR mice in a model of infection by *Cryptosporidium parvum*: the APOE4-TR mice had faster recovery than E3 for intestinal inflammatory responses and mucosal damage (Azevedo et al., 2014). The improved gastrointestinal health with *APOE4* relative to *APOE2* in mice and humans may reflect, in part, an increase in the relative abundance of Lactobacillaceae (Parikh et al., 2020). Lactobacillus has been associated with improved gut health with regards to Cryptosporidium or fungal infections and gut health (Di Cerbo et al., 2016).

### APOE Genotype and the Vascular System

*APOE4* is associated with greater levels of atherosclerosis, potentially through increased LDL-C levels from defective VLDL remnant clearance as described above. Correspondingly, *APOE4* carriers have shown a higher incidence of ischemic heart disease (Xu et al., 2016). The increased use of statins may have attenuated this adverse impact of *APOE4* (Nieminen et al., 2008).

There is evidence supporting BBB breakdown in older *APOE4* carriers. In APOE-TR models, activation of the cyclophilin A (CypA)–matrix metalloproteinase 9 (MMP-9) pathway leads to enzymatic degradation of the BBB tight junction and basement membrane proteins, resulting in BBB breakdown followed by neuronal uptake of multiple blood-derived neurotoxic proteins (e.g., thrombin, fibrin), perivascular deposition of erythrocyte-derived hemosiderin, and microvascular and cerebral blood flow reductions. The vascular defects in APOE4-TR mice appear to precede neuronal dysfunction and may initiate neurodegenerative changes. Also, this study showed that the astrocyte secreted apoE3 and apoE2, but not apoE4, suppressed the CypA–MMP-9 pathway in pericytes *via* low-density lipoprotein receptor-related protein 1 (LRP1; Bell et al., 2012). In humans, postmortem brain tissue analysis support BBB breakdown in patients with AD, which is more pronounced in *APOE4* carriers compared with *APOE3* or *APOE2* (Zipser et al., 2007). The CSF plasma albumin quotient, a marker of BBB breakdown, is greater in older (above 65) cognitively normal *APOE4* carriers compared to persons carrying the other genotypes (Halliday et al., 2013). Ongoing studies are examining whether more subtle vascular changes at the BBB appear in younger cognitively normal *APOE4* carriers.

### APOE Genotype and the Brain

Among its pleiotropic effects on aging, *APOE4*’s strongest effects are arguably on the brain. *APOE4* is the strongest genetic risk factor for late-onset AD, with a correspondingly earlier accumulation of amyloid plaques and neurofibrillary tangles (Verghese et al., 2013; Jansen et al., 2015). However, populations differ in *APOE4*’s risk-effect, which is lower for Latino and African Americans than Caucasians (Farrer et al., 1997). Population differences in *APOE* alleles are discussed below.

Brain development is directly influenced by *APOE* alleles. In the Pediatric Imaging Neurocognition and Genetics Study of 1,187 healthy children, *APOE4* carriers had thinner temporal cortex, smaller hippocampus in correlation with weaker executive functions (Chang et al., 2016). This study confirmed the early findings of Shaw et al. (2007). Because cortical thinning is an AD risk factor (Konishi et al., 2018), these neurodevelopmental effects of *APOE4* anticipate the accelerated trajectory of cognitive aging. At the cellular level, dendritic spine structure also differs: *APOE4* carriers had thinner dendritic spin heads inversely proportionate to the levels of NFT in the frontal cortex (Braak score; Boros et al., 2019). APOE4-TR mice have fewer dendritic spines with lower spine volume than the E3 (Ji et al., 2003; Sun et al., 2017). Correspondingly, the differentiation of adult neural stem cells (NSC) into hippocampal dentate granule neurons had less total dendritic length and complexity; However, NSC proliferation did not differ by *APOE* allele (Tensaouti et al., 2018).

*APOE4* is associated with glucose hypometabolism in the brain of older adults (Wolf et al., 2013), and with both markers of astrocytosis and microgliosis (Fernandez et al., 2019). In the Mayo Clinic study, older *APOE4* carriers demonstrate greater glucose hypometabolism in AD-affected brain areas than non-carriers. These changes are not associated with fibrillary amyloid detected by PET imaging (Knopman et al., 2014), but smaller aggregates and oligomers may still be a factor. In the subgroup of participants between the ages of 30 and 60 years from this study (*n* = 62), there were no significant regional differences between *APOE4* carriers and noncarriers (Knopman et al., 2014). The effect of *APOE4* on glucose hypometabolism in younger (middle aged) cognitively normal adults is more evident in *APOE4* homozygotes than heterozygotes (Mosconi et al., 2004; Reiman et al., 2004). Proposed mechanisms include changes in apoE protein expression levels, qualitative differences in apoE proteins (for example, aggregated vs. lipidated ApoE), a direct effect of apoE on nuclear transcription, and complex interactions with Aβ (Fernandez et al., 2019). Another mechanism involves apoE’s effect on endosomal trafficking. Brain endosomes are enlarged decades before the onset of cognitive decline in *APOE4*, particularly in pyramidal neurons in the inferior frontal lobe (Cataldo et al., 2000; Nixon, 2005). APOE-TR mice corroborate these postmortem findings, with enlarged endosomes and increased endosomal trafficking proteins in APOE4 vs. APOE3-TR brains in the entorhinal cortex area of APOE-TR mice (Nuriel et al., 2017b; Peng et al., 2019).

Since apoE interacts with several receptors as it traffics into the endosomes of neurons and astrocytes, endosomal trafficking affects several pathways relevant to AD pathogenesis. For example, apoE forms complexes with the neuronal IR, shifting it from the plasma membrane to endosomal compartments contributing to the phenotype of brain IR (Zhao et al., 2017). ApoE4 complexes with synaptic receptors reducing neuronal surface expression of ApoER2, as well as NMDA and AMPA receptors by sequestration in intracellular compartments, causing reduced enhancement by Reelin of glutamate synapses (Chen et al., 2010). In astrocytes, apoE complexes with LRP1. Reduced recycling of LRP1 to the plasma membrane reduces the ability of astrocytes to degrade Aβ peptides (Prasad and Rao, 2018) and provides one mechanism for the increased formation of amyloid plaques that are associated with *APOE4*. We have shown that *APOE4* can form complexes with ABCA1 in astrocytes, trapping ABCA1 in late endosomes (Rawat et al., 2019). Lower ABCA1 activity is associated with lower cholesterol transport and an increase in intracellular and plasma membrane cholesterol content. An increase in neuronal membrane cholesterol composition affects APP processing and increases TLR-4 dependent inflammasome activation. Increased cellular cholesterol in microglia limits its ability to degrade Aβ peptides (Lee et al., 2012). Taken together, reduced recycling of ABCA1, the IR, LRP1, ApoER2, synaptic receptors and other proteins complexed with the apoE4 protein provide one explanation for the accelerated brain aging phenotype observed in *APOE4* carriers.

## *APOE* Genotype and the Chromosome 19q13 Gene Cluster

Other genes linked to *APOE* on Chromosome 19 must be considered for the association of *APOE4* aging and disease. The immediate neighbor of *ApoE* is *TOMM40*, which encodes a mitochondrial transport protein. Variants of TOMM40 with intronic poly-T tracts of varying length (*TOMM 523*) are associated with AD (Roses et al., 2010). Genetic variants of the adjacent TOMM40 and *APOE* on Ch19q13.3 are independently and additively associated with dementia risk in Caucasian and African-American populations (Yu et al., 2017). Moreover, alleles of *APOE* and *TOMM40* modify many aspects of brain aging that arise before clinical-grade AD, including cognitive processing and cortical atrophy, loss of myelin, and cerebral microbleeds (Johnson et al., 2011; Lyall et al., 2014).

The *APOE4* rs429358 polymorphism was associated with higher BMI at later ages more than for younger ages, which may contribute to late-life specific increased risk of AD by regulating body fat, as discussed above. This association is consistent with increased risk of AD with age in the general population and higher risk or underweight subjects to develop AD in old age (Joo et al., 2018). There are additive effects of rs2075650 and rs157580 TOMM40 variants and rs429358 and rs7412 *APOE* variants coding the ε2/ε3/ε4 polymorphism on BMI in age-aggregated and age-stratified cohort-specific and cohort pooled analysis of 27,863 Caucasians aged 20–100 years from seven longitudinal studies (Kulminski et al., 2019).

Recently, Kulminski et al. (2019, 2020) and Wolters et al. (2019) documented new AD risk variants in 11 more genes in 19q13.3 (Table 3) Together with its AD-associated genes, the 19q13.3 locus includes more than 50 other genes with diverse functions (Table 3), including lipid metabolism and transport (ApoC1), inflammatory mediators (NFkB, PVRL2), reproductive hormones (luteinizing hormone), and transcription factors (NFkB, zinc finger). While many of these genes do not have reported AD associations, we include them because of the possibilities of co-regulation.

**Table 3:** Chromosome19q13.13.1–13.2.

		AD-association
APOE4/q13.31	APOE4 associated more with apoB lipoproteins	Roses et al. (2010) and Kulminski et al. (2019)
APOC1/q13.32	Inhibits CETP; all lipoprotein particles VLDL;	Kulminski et al. (2019) and Zhou et al. (2019)
APOEC1P/q13.32	Pseudogene	Kulminski et al. (2019)
APOC2/q13.32	activates LP lipase for triglyceride hydrolysis	Kulminski et al. (2019)
APOC4/q13.32	VLDL	Kulminski et al. (2019)
BCAM/q13.32	basal cell adhesion molecule	Kulminski et al. (2019)
BCL3/Q13.32	B cell leukemia protein 3, transcription factor	Kulminski et al. (2019)
CGB/q13.32	chorionic gonadotrophin	
CLPTM1/q13.32	cleft lip and palate transmembrane factor 1	Kulminski et al. (2019)
CYP2A/q13.2	cytochrome P450	
C5aR1/q13.3–13.4	complement factor 5a receptor 1	
FOXA3/q13.2–13.4	forkhead box transcription factor	
IGFL1–4/q13.32	IGF-like family	
IRF2BP1/q13.32	Interferon regulatory factor 2-binding protein 1, cotranscription factor	
LHB/q13.32	luteinizing hormone beta peptide	
NECTIN2/q13.32	herpes receptor (HHV-1); also PVRLl2	Kulminski et al. (2019) and Zhou et al. (2019)
NTF4/q13.3	neurotrophin	
OPA3/q13.32	outer mitochondrial membrane	
PVRL2/q13.32	poliovirus, receptor-related protein; nectin 2	Kulminski et al. (2019) and Zhou et al. (2019)
RELB/q13.32	NFkB subunit, transcription factor	
TOMM40/q13.32	translocase of outer mitochondrial membrane 40 kDa	Roses et al. (2010) and Kulminski et al. (2019)
TGFβ1/q13.2	Transforming growth factor β1	
ZNF/q13.2	Zinc finger transcription factors, > 20	

*http://compgen.rutgers.edu/scr19q13.11-19q13.33_kgenes.shtml*.

Several Ch19q13 genes are co-regulated at a transcriptional level: *ApoE-TOMM40-ApoC1* showed parallel responses to PPARγ, a ligand-activated transcription factor, and have promotor DNA binding domains for PPARγ (Subramanian et al., 2017).

Besides its role as a lipoprotein, there is evidence that the apoE protein is a direct transcriptional regulator (Theendakara et al., 2016, 2017, 2018). In their initial study (Theendakara et al., 2016), chromatin pull-down (ChIP) associated apoE with about 3,000 genes, and about half of these were restricted to apoE4, but not apoE3. Promoters of four genes were transcriptionally repressed by apoE4: ADNP (Ch20), COMMD6 (Ch13), MADD (Ch11), and SirT1 (Ch10). ApoE was bound to the SirT1 promoter sequence cagcctccgcccgccacgtgacccgtagtg, with a Kd of 3 nM.

### Ethnic Differences in the Associations of APOE4 With AD Risk

*APOE* allele frequencies may vary widely within regions, illustrated by the 3-fold gradient of *APOE4* from Nordic to Mediterranean countries in Europe, e.g., Finland and Sweden (22%) vs. Italy and Spain (8%; Lucotte et al., 1997; Mastana et al., 1998). Basques in Spain have even lower *APOE4* (6%). Although *APOE4* also increases the risk of AD and CVD in these populations, there is less correspondence of *APOE4* prevalence with lifespans in these national populations: Finland, 81.4 years; Sweden 82.7 years vs. Italy 83.7 years and Spain 83.1 years. Within countries, however, subpopulations differ importantly in the strength of *APOE4* as an AD risk factor.

Ethnicities differ in AD associations with *APOE4*, which is a 30–50% weaker association for African Americans and Latinos than Caucasians (Tang et al., 1996; Farrer et al., 1997; Rajabli et al., 2018). For Latinos with AD, *APOE4* was 30% less frequent than Caucasians in Texas: 38% (*N* = 35) vs. 60% (*N* = 160; O’Bryant et al., 2013), consistent with findings from California (Haan et al., 2003) and Northern Manhatten (Tang et al., 1996). Myriad environmental and lifestyle factors in the AD exposome may interact with the *APOE* alleles (Babulal et al., 2019; Finch and Kulminski, 2019).

Additionally, neighboring genes to *APOE* on chromosome 19.3 interact with *APOE4*. Its nearest neighbor, TOMM40, has variants of intronic poly-T repeat lengths that differ by ethnicity as briefly noted above. Several population studies showed differing AD risk for APOE ε4-TOMM40 ‘523 haplotypes defined by poly-T length haplotypes: “short (‘523S, 19 nt)” and “long” (“523L” > 30 nt). Caucasian ApoE3/3 carriers with AD are predominantly ‘523L (Roses et al., 2014; Yu et al., 2017). The older Caucasians and African Americans differed widely in the frequency of ‘523. Caucasians (*N* = 1,848) had almost entirely E4-‘523L (94%), with <1% ‘523S; contrastingly, African-Americans (*N* = 540) had only 48% ‘523S, and 1.1% ’523L. For Caucasians, each copy of ApoE4 and ‘523L doubled AD risk, with allele dose effects. For African-Americans, the absence of ‘523L in *APOE4* carriers weakened the impact of *APOE4*: those without had weaker risk effect than the few (1%) with E4-‘523L; E4 plus ‘523L increased AD risk. Much less is known of other populations. The Japanese E3-‘523S is less frequent than in Caucasians, whereas the E4-‘523S is common as for African Americans (Nishimura et al., 2017).

The cause of the *APOE* heterogeneity in the AD risk effect is obscure. The major possibilities are genetic variation local to the *APOE* region that differs among populations. We must also consider the myriad environmental, lifestyle, and cultural factors correlated with ancestry. Rajabli et al. (2018) analyzed *APOE* genotypes and genome-wide array data in several African American and Puerto Rican populations: [1,766 African American and 220 Puerto Rican individuals with late-onset AD, and 3,730 African American and 169 Puerto Rican cognitively healthy individuals (>65 years)]. The analysis indicated the importance of ancestry-specific genetic factors near the *APOE* locus rather than non-genetic ethnic, cultural, and environmental factors by the lower risk effect in the *APOE4* allele. The linkage disequilibrium (LD) showed that the roles of the ε4- and ε2- coding SNPs in AD were dependent on the other SNPs in this locus. Differences between white and nonwhite populations in LD structure and changes in LD between the AD-affected and -unaffected subjects may explain differences in risks of AD for these alleles in these populations (Kulminski et al., 2020).

## The Response of *APOE4* Carriers to Dietary and Lifestyle Interventions

We have identified important factors that can inform the choice of future dietary and pharmacological interventions designed to mitigate the aging effects of *APOE4*. The first is the co-regulation of *APOE-TOMM40-APOC1* locus by PPARγ. The second is related to the effect of *APOE4* on brain energy preference, including how weight loss later in life increases the risk of cognitive decline among *APOE4* carriers.

### The Resistance of APOE4 Carriers to Drugs Targeting the PPAR-LXR/RXR-APOE System

The challenge with the blunted induction of PPARγ pathways in *APOE4* carriers is clearly illustrated in several clinical trials using PPARγ or RXR agonists for cognitive and AD-related outcomes. In one randomized clinical trial, 511 subjects with mild-to-moderate AD were randomized to groups receiving placebo or 2, 4, or 8 mg rosiglitazone (PPARγ agonist) for 24 weeks (Risner et al., 2006). At week 24, the subjects were evaluated for mean change from baseline in the Alzheimer’s Disease Assessment Scale–Cognitive subscale (ADAS-Cog) battery and Clinician’s Interview-Based Impression of Change Plus Caregiver Input global scores. Rosiglitazone at any dose did not significantly alter cognition by these tests. However, *APOE4* non-carriers showed (*n* = 323) significant improvement in ADAS-Cog results at the highest dose of 8 mg rosiglitazone. No improvement and some decline in mental acuity were observed in *APOE4* positive subjects.

The TOMMORROW secondary AD prevention trial (NCT01931566) tested whether pioglitazone (PPARγ agonist) would prevent mild cognitive impairment (MCI) in asymptomatic people at genetic risk for AD (Burns et al., 2019). It was stopped early after a futility analysis gave it only a 15% chance of success. The trial enrolled 3, 494 cognitively normal participants at risk of developing cognitive impairment (CI) based on an algorithm that weighed their *APOE* and TOMM40 genotypes and ages. The primary endpoint was progression to MCI. Time to progression was the same in both pioglitazone and placebo groups assessed out to 36 months. The cognitive composite battery score increased over time in both groups, while ADCS-ADL scores remained constant. More than 60% of people in the high-risk group had *APOE4*. The analysis is underway to understand the *APOE* genotype effect on the response to the intervention.

Another clinical trial that targeted the RXR transcription pathway was Beat-AD. Beat-AD was a double-blind, randomized, placebo-controlled, parallel-group study that examined the effect of a single dose (300 mg/day) of bexarotene in 20 participants with early AD (Cummings et al., 2016). The primary outcome (brain amyloid index) did not change after 1 month of treatment. However, a preplanned secondary analysis revealed a decrease in the brain amyloid index in *APOE4* non-carriers. These changes were correlated with increased plasma Aβ levels, and suggested a role for bexarotene in non-*APOE4* carriers (Cummings et al., 2016). In summary, three trials using PPARγ or RXR agonists were not effective in slowing the progression to MCI or AD. Two out of these three trials suggest an *APOE* genotype effect: *APOE4* blunted the response to these interventions on cognitive and AD biomarker outcomes.

### APOE4 Brain Fuel Preferences and Response to Diet

The lower brain glucose metabolism and the increased mitochondrial oxidation of PUFAs in older *APOE4* carriers suggest a role for dietary fat as brain fuel. In a small pilot trial, older *APOE4* carriers with cognitive impairment (CI) appeared to respond to an increase in dietary fat intake for cognitive functions. In this study, 46 older adults with either CI or normal cognition (NC) ingested a LOW (25% total fat) and a HIGH-fat meal (50% total fat) in an acute and blinded random fashion. Acute high-fat feeding improved measures of cognition and plasma AD biomarkers in E4 carriers, but worsened these biomarkers in E4 noncarriers (Hanson et al., 2015). These findings were driven by the CI and not the NC group. There were no differences in LDL-C after this acute fat intervention. Findings from this pilot trial, however, need to be replicated in a larger study, but they underscore the differential response by both *APOE* genotype and cognitive state to high-fat ingestion. These findings may be counter-intuitive given that *APOE4* carriers have higher LDL-C levels and that saturated fat intake can modestly increase levels of LDL-C. Interestingly, *APOE4* also modulates the effect of switching from a high-fat diet to a low-fat diet on plasma cholesterol levels: *APOE4* carriers who switched from a high-fat diet to low fat and low glycemic index high carb diet had greater reductions in LDL-C (Griffin et al., 2018).

Older *APOE4* carriers with CI also show resistance to improvement from a ketogenic diet. Two interventions demonstrated that *APOE4* carriers do not benefit from a ketogenic diet (Reger et al., 2004; Henderson et al., 2009). In one of these interventions (Henderson et al., 2009), 152 participants with mild AD were randomized to AC-1202 to rapidly elevate serum ketone bodies or placebo. The intervention resulted in modest differences in ADAS-Cog scores compared to the placebo. However, the effects were only seen in *APOE4* negative subjects who were compliant with the intervention. Understanding the type of diet that the brain of older *APOE4* carriers utilize as fuel would be a priority for future studies.

### The Cognitive Vulnerability of Older APOE4 Carriers to Weight Loss

Clinical trial evidence suggests that *APOE4* increases cognitive vulnerability to weight loss. The Look AHEAD trial was a single-blinded, randomized, controlled trial that recruited 5,145 individuals who were overweight or obese and had type 2 diabetes. Participants underwent an Intensive Lifestyle Intervention (ILI) or Diabetes Support and Education (DSE) intervention. Cognitive outcomes were assessed 10–13 years after enrollment. The intervention did not affect cognitive outcomes (Espeland et al., 2017; Rapp et al., 2017). In a subgroup analysis, we observed a significant interaction between the onset of menopause, *APOE4*, and the intervention on cognitive scores. Older postmenopausal women had worse cognitive scores in the ILI group compared with the DSE group. In contrast, younger pre- or early postmenopausal females had better cognitive scores in the ILI group compared with the DSE group. The positive effect of weight loss was only evident among *APOE4* non-carriers (Yassine et al., 2020). These findings support that weight loss in *APOE4* carriers may deprive the brain of an important source of fuel: fat stored and released from adipocytes.

### A Role for Omega-3 Enriched Diets in APOE4 Carriers

**Figure 5:**
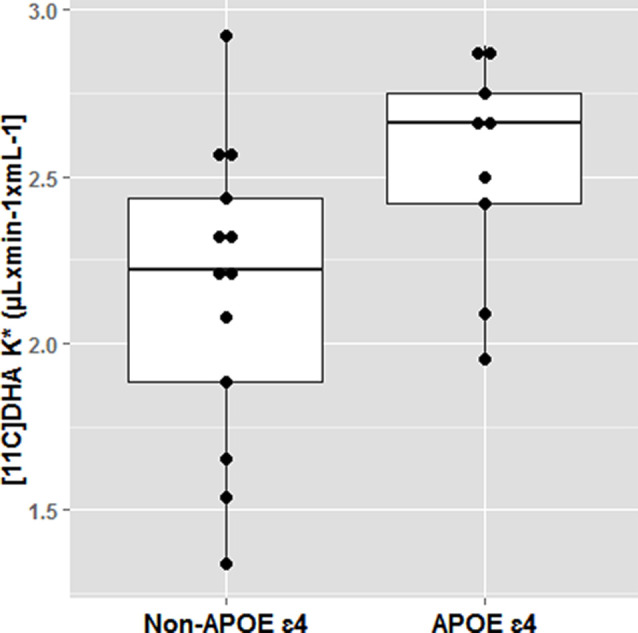
Docosahexaenoic acid (DHA) brain uptake by *APOE4* carrier state. Cognitively healthy younger *APOE4* carriers had greater brain DHA uptake using an ^11^C-DHA PET scan. *p < 0.05. Adapted from Yassine et al. (2017b).

The effect of *APOE4* on omega-3s has been demonstrated in several elegant animals and human kinetic tracer studies. Following an omega-3 deficient diet, adipose tissues in APOE4-TR mice had 40% less omega-3 than APOE3-TR mice. Human studies also confirm that *APOE4* carriers are more vulnerable to dietary omega-3 deficiency and may require long term dietary DHA consumption than non-carriers for maintaining brain DHA supply. Using PET scans, we have identified that brain DHA uptake was 20% greater in younger cognitively normal *APOE4* compared to non-carriers (mean age 35) suggesting a brain DHA deficit that is compensated with a higher plasma to brain DHA delivery (Yassine et al., 2017b; Figure 5). Since the brain does not have an efficient mechanism to store fat, any compromise in adipose ω-3 stores can affect brain delivery.

Some evidence reveals that the *APOE* genotype affects the response to ω-3 supplementation, although some of these results are inconsistent. Some observational studies do not reveal an effect of *APOE* status on the association of ω-3 with cognitive outcomes (Beydoun et al., 2007; Krüger et al., 2009; Rönnemaa et al., 2012). We reported an inverse association between low serum DHA levels and cerebral amyloidosis in older non-demented participants independent of *APOE* genotype (Yassine et al., 2016a). In some observational studies, the benefit of increased seafood or ω-3 consumption on cognition was restricted to *APOE*4 non-carriers (Huang et al., 2005; Barberger-Gateau et al., 2007; Whalley et al., 2008; Daiello, 2015), and in particular those with limited seafood intake (<1 serving/week; Huang et al., 2005; Barberger-Gateau et al., 2007). The ADCS-sponsored DHA trial reported a null effect on cognitive outcomes, but a pre-planned analysis revealed cognitive benefit (using ADAS-cog scale) in the DHA treatment arm in *APOE*4 non-carriers (Quinn et al., 2010).

In other studies, the benefit was restricted to *APOE4* carriers (Laitinen et al., 2006; van de Rest et al., 2008; Stonehouse et al., 2013; Morris et al., 2016). In two of those studies, the beneficial response in *APOE4* carriers was observed in younger participants (Stonehouse et al., 2013), mean age = 33, randomized clinical trial, and (Laitinen et al., 2006), mean age = 50, an observational cohort with 20-year follow-up. In a cross-sectional study of deceased participants from the Rush Memory and Aging Project (Morris et al., 2016), participants were dementia-free at study entry and underwent annual clinical neurological evaluations and brain autopsy at death with a mean follow-up duration of 8 years. Individuals who were *APOE4* carriers and consumed at least 1 seafood meal per week or had higher intakes of long-chain ω-3 fatty acids had less AD neuropathology post-mortem compared with those who consumed lower amounts.

**Figure 6:**
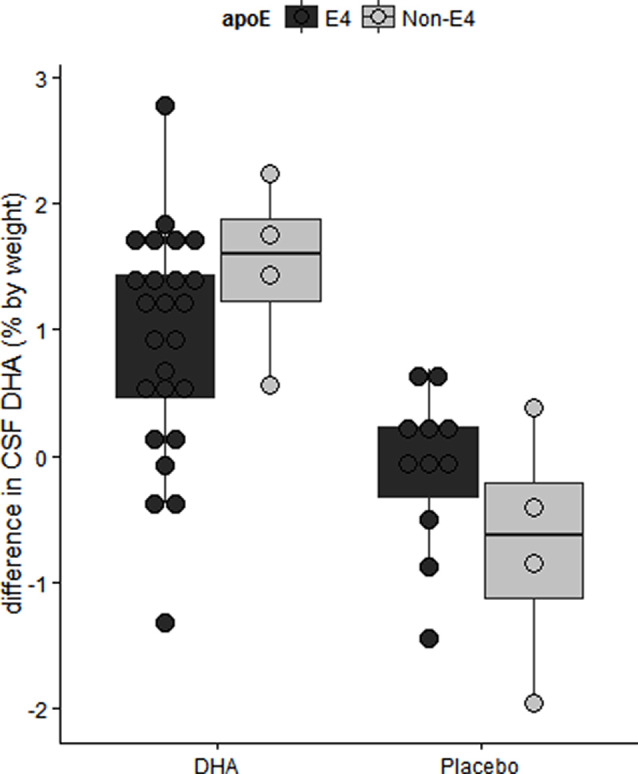
The difference in CSF DHA levels by intervention arm in the ADCS sponsored DHA clinical trial. Older *APOE4* carriers with mild AD had lower CSF DHA levels after 18 months of DHA supplementation. Adapted from Yassine et al. (2016b).

We have reported in the ADCS-sponsored DHA clinical trial that baseline CSF DHA levels were lower in *APOE4* carriers compared with *APOE2* carriers (Yassine et al., 2016b). After treatment, we observed lower DHA levels in persons with more advanced brain disease as determined by the lowest tertile of CSF Aβ42 levels (Figure 6; Yassine et al., 2016b). *APOE4* changes also included a lower increase in plasma DHA and eicosapentaenoic acid (EPA) ratio to arachidonic acid (AA) after supplementation (Tomaszewski et al., 2020). These findings agree with preclinical studies in 13-month-old APOE-TR mice, where brain DHA levels were lower in *APOE4*-TR mice compared with *APOE2*-TR mice (Vandal et al., 2014). Accordingly, we proposed a complex interaction between *APOE*4 status and disease stage, such that the response to ω-3 supplementation in *APOE*4 c*arriers* depends on whether supplementation precedes the onset of neurodegeneration (Yassine et al., 2017a), and requires high dose supplementation and a long term intervention.

Among the best-studied diets for AD prevention is the Mediterranean diet. This diet differs by Mediterranean countries but generally characterized especially by high consumption of vegetables, polyunsaturated fat (fish and nuts), olive oil, and moderate consumption of protein. Most studies have demonstrated cognitive or AD biomarker benefits of the Mediterranean diet despite modest effects on weight (Tsivgoulis et al., 2013; Ngandu et al., 2015; Pelletier et al., 2015). The Finger trial included *APOE* genotype as a controlling factor. In this trial, a multicomponent intervention study involving 2 years of increased fish consumption, fruits, and vegetables together with exercise and brain training resulted in a modest improvement in cognitive outcomes (Ngandu et al., 2015). A subgroup analysis revealed that *APOE4* carriers had a 2.6 fold greater benefit on the total composite NTB outcome from this intervention (Solomon et al., 2018), although the interaction between *APOE* genotype and intervention arm on cognition was not statistically significant.

The Multi-domain Alzheimer Prevention Trial (MAPT) was a three-year intervention trial designed to assess whether a combined intervention of cognitive stimulation, physical activity, nutrition, and supplementation with omega-3 polyunsaturated fatty acids could slow cognitive decline in a population of older adults at risk for AD. The results of the study, published in 2017, failed to demonstrate a significant slowing of cognitive decline during the 3-year study period, although subgroup analyses suggested possible (and modest) benefits for individuals with elevated brain amyloid accumulation and those who were carriers of the *APOE4* allele (Andrieu et al., 2017).

## Designing Future Interventions

Given the complex interaction of *APOE4* with several genetic and environmental factors that shape the response to diet, we propose considering novel designs for nutritional clinical trials aiming to improve cognitive outcomes in *APOE4* carriers.

1) Specific recruitment and stratification by *APOE4* carrier status, with sample sizes sufficient to allow detecting an *APOE4* by treatment interaction
2) Utilization of brain-specific biomarkers to predict the response of intervention before conducting large and extensive trials. For example, given the greater DHA brain uptake in *APOE4* carriers shown in Figure 5 (Yassine et al., 2017b), the efficacy of PUFA enriched diets can be guided by change in brain DHA PET uptake. Other imaging modalities such as ketone, glucose, AA, and other PET imaging modalities can guide a choice of specific diets. There is an urgent need for less invasive brain-specific nutrient biomarker panels to guide larger trials.
3) Since the risk of disease in *APOE4* is affected by complex interactions, trials would need to include other risk factors (sex, race, obesity, menopausal state, or coinheritance of other gene variants) for resolution of both the *APOE4* and the treatment effects.
4) *APOE4* disease risk appears to start at a very early age. New cognitive outcomes are needed to identify the earliest stages of disease for preventive measures before the onset of irreversible neurodegenerative changes.
5) Given the blunted PPARγ response in *APOE4* carriers, we should consider combining pharmacotherapy to restore the PPARγ signaling response in *APOE4* carriers to weight loss with exercise interventions.
6) Development of selective PPARγ signaling molecules that uncouple the co-expression of bioenergetic/insulin-sensitizing PPARγ program from APOE expression may be useful for drug development
7) Enhancing apoE recycling by reducing apoE aggregation (for example through increasing HDL3 or by ABCA1 agonists) may have downstream benefits on cellular energy preferences and the response to the diet on the brain.

## Summary

In summary, carrying the *APOE4* allele poses an increased risk of neurodegenerative, cerebrovascular, and CVD with aging that is race and sex-specific. *APOE4* continues to dazzle the scientific community and represents both an opportunity and a challenge. *APOE4* affects cellular preferences for energy during aging with preclinical and clinical evidence indicating a shift from glucose to PUFA fatty acids as a source of energy, increasing the susceptibility of the brain to disease when ω-3 intake is restricted. However, the effects of *APOE4* on aging are complex and differ by sex, race, and the environment. The gene by environment interactions on the predisposition of *APOE4* to disease requires more sophisticated interventions. *APOE* genotype has a complex relationship with inflammation that differs by race and region. *APOE4* carriers with markers of chronic inflammation appear to be protected in some studies against infections, but possess a greater risk of dementia in others. Therefore, a greater understanding of how the environment affects the susceptibility to disease in some but not all *APOE4* carriers requires more targeted and personalized approaches. Over the next decades, *APOE* personalized strategies will better guide our approach in reclassifying and targeted managing of *APOE4*-associated aging diseases.

## Author Contributions

HY reviewed the literature on APOE and diet. CF reviewed the literature covering APOE genetics and sex. Both authors reviewed basic apoE biology.

## Conflict of Interest

The authors declare that the research was conducted in the absence of any commercial or financial relationships that could be construed as a potential conflict of interest.
